# The enigma of the dog mummy from Ancient Egypt and the origin of ‘*Rhipicephalus sanguineus*’

**DOI:** 10.1186/1756-3305-7-2

**Published:** 2014-01-20

**Authors:** Domenico Otranto, Jean-Bernard Huchet, Alessio Giannelli, Cecile Callou, Filipe Dantas-Torres

**Affiliations:** 1 Department of Veterinary Medicine, University of Bari, Valenzano, Bari, Italy; 2 UMR 7209 du CNRS, Archéozoologie, Archéobotanique, Muséum National d’Histoire Naturelle, Paris, France; 3 Department of Immunology, Aggeu Magalhães Research Centre, Oswaldo Cruz Foundation, Recife, Pernambuco 50670420, Brazil

**Keywords:** Tick, *Rhipicephalus sanguineus*, Dog mummy, Archeoparasitology, Origins

## Abstract

**Background:**

Ticks belonging to the *Rhipicephalus sanguineus* group are amongst the most important vectors of pathogenic microorganisms to dogs and humans. However, the taxonomy of this species group is still the subject of debate, especially because there is no type specimen or reliable morphological description for *Rhipicephalus sanguineus* sensu stricto. Recently, a comprehensive morphological and genetic study on representative tick specimens from Europe, Africa, Americas, and Oceania, revealed the existence of at least four morphologically and genetically distinct species under the name ‘*R. sanguineus*’ infesting dogs from different countries.

**Methods:**

Herein, we examined morphologically tick specimens retrieved on a dog mummy from Ancient Egypt (*ca.* 1^st^ century – 4^th^ century A.D.). The dog mummy and associated ticks were found during an archaeological expedition conducted in El Deir.

**Results:**

Scanning electron micrographs allowed us to assess their identity as belonging to the *R. sanguineus* group. In addition on the basis of the scutal punctation pattern, spiracular plates, width of dorsal tail of spiracular plates relative to the adjacent festoon, female genital aperture, male adanal plates and accessory shields, these ticks were tentatively identified as *Rhipicephalus* sp. II (=temperate species).

**Conclusions:**

It can be concluded that *R. sanguineus* group ticks have infested dogs living in the Mediterranean region since ancient times. This finding represents the oldest record of ticks on any animal species and adds a new piece in the complex puzzle regarding tick parasitism on dogs and humans and their role as vectors of pathogens.

## Background

Among domestic animals, dogs have always been alongside humans while hunting, migrating around the globe, and even while exploring the moon. Indeed, for their devotion to their owners and friendly behaviour, dogs have represented the most common pet for humankind throughout their history. Meanwhile, as good friends, dogs share many things with humans, including zoonotic endo- and ecto-parasites [1,2]. Amongst the arthropod parasites of both dogs and humans, the brown dog tick *Rhipicephalus sanguineus* (Latreille, 1806) is an efficient vector of a diverse group of pathogens [3]. However, in spite of being well-studied, the taxonomy of *R. sanguineus* is largely debated among scientists, because there is no type specimen or reliable morphological description for this species [4]. Therefore, while *R. sanguineus* could be regarded as a *nomen nudum*, this tick is still listed as a valid taxon [5].

During the last century, *R. sanguineus* was placed in synonymy with many species [6,7], whereas others, morphologically similar, were described and ranked within the so-called *R. sanguineus* group, whose definition and number of species is also arguable [7-9]. Several authors have endeavoured to study this group of ticks using both morphological and molecular tools [4,10-16]. Recently, a comprehensive study was undertaken on representative tick specimens belonging to the *R. sanguineus* group from 17 countries in Europe, Africa, Americas, and Oceania [4]. Morphological and molecular analyses revealed the existence of at least four integrated operational taxonomic units (i.e., *R. sanguineus* sensu lato, *Rhipicephalus* sp. I, *Rhipicephalus* sp. II, and *Rhipicephalus* sp. III) under the name ‘*R. sanguineus*’ [4]. Nonetheless, in the absence of a consensus on the identity of *R. sanguineus* sensu stricto, the taxonomical status and actual distribution of these species remains enigmatic.

As for any investigational research, parasitologists need to look for any clue (e.g., morphological, molecular, biological, ecological evidence) to address their questions or hypotheses. Of great interest is to understand when and how parasites developed in animal and human populations. Under these circumstances, archeoparasitology not only investigates the causes of the death of the hosts infected by parasites [17], but also how they moved from one area to another, along with animals and humans during historical migrations [18]. Oddly enough, studies in the field of archeoparasitology have been mainly focussed on protozoa and helminths in coprolites, intestinal contents or latrine deposits [19-23]. In contrast, despite the tough chitinous exoskeleton of arthropods, a relatively low number of archaeoparasitological surveys are available for ectoparasites [24-29], probably because of their location on the host coat, therefore more exposed to the outdoor environment. In addition, archeoparasitological studies on pets are limited to the retrieval of lice from cats [30] and dogs [28,31]. To the best of our knowledge, the only possible iconographic illustration of ticks from Ancient Egypt is constituted by a tomb painting from ancient Thebes (Dra Abu el-Naga, Western Thebes, ca. 1473-1458 B.C.), which displays a hyaena-like animal with excrescences within the ear that were supposed to be ticks [32].

The recent finding of well-mummified dogs from Ancient Egypt in an archaeological expedition conducted in El Deir led to the retrieval of some specimens of ixodid ticks and louse flies on a young dog [33]*.* Considering the current debate on the taxonomy of this tick species, we decided to carefully re-examine these ticks morphologically, using scanning electron micrographs to assess whether they fit with any of the species illustrated in ref. 4.

## Methods

The dog mummy was found in a tomb surrounding a Roman fortress in El Deir [33]. This archaeological site, located 30 km northeast of the town of Kharga (Kharga Oasis, Egypt) at the bottom of Gebel Umm el Ghanayim, has been excavated since 1988 [34-37] in an area including an important agricultural inhabited region, which existed at least from the Ptolemaic period (332–30 B.C.). The necropolises are dated back to a period from the 4^th^ century B.C. to the 5^th^ century A.D. Although this ancient village has not been precisely located, a number of tombs were spread over this area, and, most of them were spoiled over the centuries.

Some anatomical investigations were conducted on many rests of dogs [38], but the mummy of a young dog from tomb P5 was the only well preserved specimen. This mummy displayed a massive infestation by ectoparasites with 61 ticks found firmly attached to the animal coat, mostly in the right ear. Additionally, a single specimen of the louse fly *Hippobosca longipennis*, as well as more than two hundred puparia of sarcosaprophagous flies of the families Sarcophagidae and Calliphoridae, were also identified (for more details, see ref. 33).

Of the 61 ticks collected, only five specimens (i.e., three males and two females) were better preserved and therefore herein morphologically studied. Ticks were examined at El Deir archaeological site, using a portable scanning electron microscope (SEM) (Nikon JCM-5000, JEOL NeoScope at 10 kV), without metallization (metal coating). Micrographs were taken from the five specimens and several characters (see below) were examined and/or measured.

All images were carefully evaluated and taxonomically relevant characters for the differentiation of ticks belonging to the *R. sanguineus* group [4,8,39] assessed. In particular, the following characters were studied: idiosoma, dorsal *scutum*, angles of *basis capituli*, female porose areas, female genital opening, spiracular plates, lateral and postmediam grooves, cervical pits, cervical fields, internal and external cervical margins, marginal lines, male adanal plates, accessory plates, and male caudal process. Measurements were not taken due to the low number of specimens available and their preservation status. Moreover, no tick specimen or parts of it were available for molecular analysis.

## Results

All ticks were unengorged except for one female, and they were identified as belonging to the genus *Rhipicephalus,* based on the following general characters: eyes present, anal groove posterior to anus, *basis capituli* hexagonal in shape, palpi short, *coxae* I deeply cleft, spiracular plates comma-shaped, and male adanal plates and accessory shields present.

**Figure 1:**
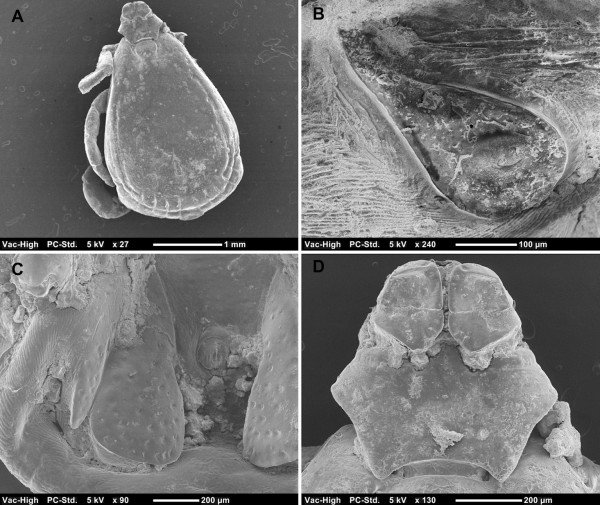
***Rhipicephalus sanguineus *group male.** Dorsal *scutum* with small punctations scattered over the posterior portion and larger punctations on the scapular region **(A)**; spiracular plate with narrow dorsal tail **(B)**; adanal plate large at base and accessory shield sharply pointed **(C)**; *basis capituli* hexagonal), dorsal view **(D)**.

Males presented the following characters: small punctations scattered over the posterior portion of dorsal *scutum*; larger punctations on the scapular region; marginal groove deep and marked by medium-size punctations (Figure 1A); posteromedian groove distinctly elongated; lateral grooves circular in shape; spiracular plates elongated, and with a narrow dorsal tail (less than half of the adjacent festoon) (Figure 1B); adanal plates large at basis (not sickle-shaped); accessory shields sharply pointed (Figure 1C); caudal process present; posteromedian spur on *coxa* I longer than the posterolateral spur; angles of *basis capituli* in anterior third of its length (Figure 1D).

**Figure 2:**
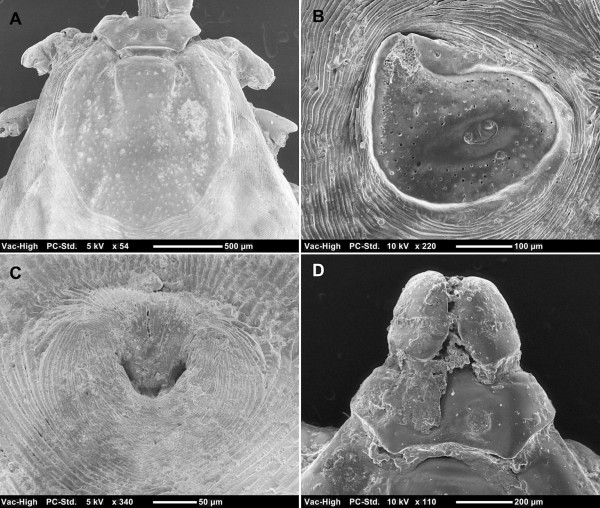
***Rhipicephalus sanguineus *group female.** Dorsal *scutum* shield-shaped, with sinuous posterior margin **(A)**; spiracular plate with narrow dorsal tail **(B)**; genital aperture broadly U-shaped **(C)**; *basis capituli* hexagonal, dorsal view **(D)**.

Females presented the following characters: dorsal *scutum* shield-shaped, with sinuous posterior margin (Figure 2A); outer edge of cervical grooves clearly defined either by slope or punctations; dorsal tail of spiracular plate narrow (Figure 2B); genital aperture broadly U-shaped (Figure 2C); posteromedian spur on *coxa* I longer than the posterolateral spur; angles of *basis capituli* at about mid-length; porose areas small, rounded and well separated (Figure 2D).

Based on these key characters, all ticks were identified as belonging to the *R. sanguineus* group. Furthermore, on the basis of the scutal punctation pattern, spiracular plates, width of dorsal tail of spiracular plates relative to the adjacent festoon, female genital aperture, male adanal plates and accessory shields, these ticks were identified as *R*. sp. II (=temperate species) (Figures 1 and 2).

## Discussion

The present study, based on a detailed morphological examination of ticks found on a dog mummy from Ancient Egypt, confirms that they belong to the genus *Rhipicephalus*. Nonetheless, the actual specific identity of these ticks is difficult to assess, mainly because of the limited number of specimens available and because of their conservation status. However, ticks herein examined were not *Rhipicephalus turanicus* or *R*. sp. III, due to the differences in scutal punctation pattern, spiracular plates and/or female genital aperture [4]. Indeed, these ticks resembled those of *R. sanguineus* sensu lato, and some specific morphological characters (e.g., female genital aperture, male adanal plates and accessory shields, scutal punctation pattern and spiracular plates of both males and females) allowed us to tentatively identify those ticks as *R*. sp. II (=temperate species or southern lineage) [4].

The high intensity of tick infestation found on this young mummified dog, along with the absence of any apparent trauma [33], may suggest that the cause of his death could be related to this massive ectoparasite infestation and/or to the infection by tick-borne pathogens. Indeed, *R. sanguineus* group ticks have been implicated as a vector of a wide range of pathogenic microorganisms to dogs (e.g., *Babesia vogeli*, *Ehrlichia canis*, *Hepatozoon canis* and *Rickettsia conorii*) some of which have zoonotic potential [3,40]. In spite of their long co-evolution with their hosts, tick-borne pathogens may cause severe disease and, eventually, the death of infected animals [41]. The dog’s death could also be attributed to a more virulent pathogen strain circulating in Ancient Egypt. For instance, although *Anaplasma platys* is often considered as a less pathogenic organism in dogs [42], virulent strains have also been associated to severe clinical disease in Israel [43]. Tick-borne pathogens commonly cause more severe disease in young individuals at their first exposure [44]. Furthermore, the sequential or simultaneous infection with more than one tick-borne pathogen may also result in the exacerbation of clinical signs and potentiation of haematological abnormalities [45]. This was recently demonstrated in young dogs coinfected with *B. vogeli* and *A. platys*, in which more severe clinical and haematological alterations were eventually recorded than in dogs with *B. vogeli* only [46]. Another possible explanation for the death of the young mummified dog could be a fatal paralytic syndrome associated with massive tick infestation. Indeed, a recent study reported neurological signs in 14 young dogs heavily infested by *R. sanguineus* group ticks, ten of which died from this condition and presented neurological signs of different degrees [47].

Although it was not possible to carry out any further parasitological investigation on other dog mummies due to their poor condition, the high number of ticks found on the studied specimen suggests that other animals were also infested. This might have been the cause of an epidemic of tick-borne disease leading to the death of many young animals. Unfortunately, the unavailability of material of ticks for molecular processing and detection of pathogens does not allow us to bring these hypotheses from the realm of speculations to reality. In the same way, the possibility that the dog died due to a viral infectious disease (common cause of sudden death in puppies) cannot be ruled out.

The so-called temperate species (=*R.* sp. II) is widespread in Mediterranean countries, such as France, Portugal, Spain and Italy [4]. Accordingly, our finding might indicate that this tick species has been present for a long time in Egypt. However, the discovery of a tick-infested dog mummy in a tomb surrounding a Roman fortress raises interesting questions on the origin of this dog and his ticks. During the Roman Empire and its colonization, which started about 270 B.C., the Mediterranean area was a theatre for relevant historical events and a hub of different cultures as well as the final destination for several populations. The intense waves of migration occurring before, during and after the Roman Empire could have contributed to the dissemination of dog ticks throughout the Mediterranean region. Indeed, the Roman Empire expanded for more than 400 years through Eurasia (from 275 B.C. to 117 A.D.) and, at its greatest extent, it colonized all the countries touching the Mediterranean sea as far as Germany and Britain (north), Turkey, Lebanon, Iran and Arabia (east) until the split in Eastern and Western sections (395 A.D.). On the other hand, because *Rhipicephalus* is typically an African tick genus, the most probable hypothesis is that *R. sanguineus* group ticks were introduced into Europe at a certain point of time, most probably with people from North Africa, soon after the collapse of the Roman Empire.

## Conclusion

The history of tick species threatening dog and human health often crosses with those of the hosts they parasitize as a part of the everyday existence of individual animals everywhere and in every time. Whether the retrieval of ticks on a mummified young dog, which succumbed around 2,500 years ago due to an obscure illness, can contribute to a better understanding of the *R. sanguineus* group or not is uncertain. Certainly, it adds a new piece in the complex puzzle regarding tick parasitism on dogs and their role as vectors of pathogens to dogs and humans.

## Competing interests

The authors declare that they have no competing interests.

## Authors’ contributions

DO and FD-T conceived the research and wrote the first draft. JBH and CC examined the dog mummy, collected the tick specimens and performed the SEM photos. FD-T, DO, and AG did the morphological study. All authors read and approved the final version of the manuscript, contributed with interpretation and revision of the manuscript.
